# Perceptions of secondary school management teams in managing pregnant learners in an urban context

**DOI:** 10.4102/hsag.v27i0.1945

**Published:** 2022-10-19

**Authors:** Phindile P. Twala, Antoinette du Preez, Tinda Rabie

**Affiliations:** 1 NuMIQ Focus Area, Faculty of Health Sciences, North-West University, Potchefstroom, South Africa

**Keywords:** secondary school, school health services, school management team, teenage pregnancy, unplanned delivery

## Abstract

**Background:**

Teenage pregnancy is a complex issue globally, which is also a challenge in South Africa. Pregnant learners are allowed by the law to attend school for the whole duration of pregnancy. Although not medically trained or equipped to handle any emergencies, the school management teams are tasked to manage these learners by the Measures of the Prevention and Management of Learner Pregnancy Policy.

**Aim:**

This study aimed to determine the school management teams’ perceptions of managing pregnant learners in urban secondary schools.

**Setting:**

This study was conducted in four secondary schools in Gauteng province, Soweto-Pimville District 10.

**Methods:**

This study employed a qualitative, descriptive design. Purposive sampling was carried out (*n* = 13), semi-structured WhatsApp video call interviews were conducted and Tesch’s data analysis steps were utilised to analyse the data.

**Results:**

Five themes emerged: Theme 1: the role of school management teams in managing pregnant learners; Theme 2: challenges of unplanned deliveries at school; Theme 3: personal thoughts of educators on managing pregnant learners; Theme 4: skills training requirements for educators to manage pregnant learners; and Theme 5: coping mechanisms for school management teams.

**Conclusion:**

Collaboration between the department of health, department of education and the department of social development is essential in the management of pregnant learners at school.

**Contribution:**

Similar studies have been conducted in various rural areas of South Africa. Limited literature was found for urban areas; therefore, the researcher is of the opinion that the findings of this study could contribute to the body of knowledge.

## Introduction

Teenage pregnancy is classified as high risk regarding maternal care as they are at an increased risk of morbidity and mortality (Jonas et al. 2016:1). Pregnancies that happen prior to the age of 20 years bear more health risks, such as pregnancy-induced hypertension, pre-term delivery because of physiological immaturity and placenta abruption, among others (Panday et al. 2009:47). According to the United Nations Educational, Scientific and Cultural Organization (UNESCO 2017), this is a global health problem. High-income countries such as the United States of America have observed a decline in teenage pregnancy rates over the past 30 years (Orimaye et al. 2021). According to Braine (2009:410), many adolescents under the age of 19 who become pregnant live in low- to middle-income countries such as South Africa (SA). As a democratic country, the *South African Constitution*, in the bill of rights 9(3) of 1996, states that the state must not discriminate against any person based on aspects such as gender, sex, pregnancy or marital status – therefore allowing pregnant learners to continue with their schooling throughout pregnancy and returning to school soon after delivery. According to a meeting report compiled by the Parliamentary Monitoring Group (PMG) (2017), it was reported that Gauteng province had the highest number of learner pregnancies in school for 2015 at 5246, followed by the Western Cape at 2891. The number of pregnant learners in Gauteng province quadrupled during the coronavirus disease 2019 (COVID-19) pandemic from 5246 to more than 23 000 between April 2020 and March 2021, and of these pregnancies, 934 were girls between ages of 10 and 14 (News24 2021). This high number is an indication that teenagers are sexually active, not using family planning methods and condoms. According to UNESCO (2017:15), poor socio-economic status is the main reason of early unwanted pregnancies in teenagers. This is supported by Leclerc-Madlala (2010:6), who indicated that, as a result of poverty, young girls enter sexual relationships with older men for transactional sex in exchange for monetary gifts, clothing and entertainment. Various policies were introduced to serve as guidelines in SA. The integrated school health policy (ISHP) was launched in 2012 by the Department of Health (DoH) in collaboration with the Department of Basic Education (DBE) with the aim of improving health and development of all school-going children, including adolescents, through Primary Health Care (PHC) re-engineering (Vlok 2014:377). The school health package covers topics such as nutrition and exercise, sexual reproductive health, menstruation, contraception and male circumcision, among others (SA, DoH & DBE 2012). The Measures of the Prevention and Management of Learner Pregnancy policy (MPMLP) was introduced as a guideline for the School Management Teams (SMTs) to assist with the management of pregnant learners at school.

### Objective

The objective of this study was to explore and describe the SMTs’ perceptions of managing pregnant learners in urban secondary schools.

## Research methods and design

### Research design

This study used a qualitative, descriptive design. This design gave a complete summary of descriptive data based on the perceptions of the SMTs in managing pregnant learners in an urban context.

### Setting

This study was conducted in Gauteng province, Soweto-Pimville District 10, in four secondary schools.

### Study population and sampling strategy

The study population comprised SMTs in four secondary schools with a high number of learner pregnancies. Purposeful sampling was used to include 13 SMT members of the selected schools. The inclusion criteria for participants were threefold: they had to be educators who are SMT members, as they are the ones tasked to manage pregnant learners at school according to the MPMLP; they had to have been an SMT member for at least one year with experience or an encounter in managing pregnant learners and had to be English literate. All educators who did not form part of the SMT were excluded.

### Data collection

Data were collected over a period of six weeks. Using an interview guide, semi-structured interviews via WhatsApp video calls were carried out in English. The interview guide included the following questions: (1) what is your role as an SMT member in the school?; (2) what is the process you follow when there is an unplanned pregnancy delivery at school?; (3) what are your thoughts on having to manage pregnant learners?; (4) what training do you think you require to manage pregnant learners at school?; and (5) what recommendations can be made to improve the management of pregnant learners? After 10 interviews, data saturation was reached, but another three were performed to ensure that no new information came forth. All interviews were recorded on a voice recorder and each interview lasted for 30–40 min.

### Data analysis

Recorded data were transcribed verbatim and the transcripts were analysed using Tesch’s eight steps in the coding process (cited by Creswell 2018:196) with the assistance of a co-coder. All the transcripts were read and re-read to obtain a general sense of the information. This process was repeated several times with the transcripts, making a list of topics and grouping relevant themes. After that, themes were formatted into columns and arranged as main themes and subthemes. Themes were thereafter abbreviated as codes. These codes were written in the relevant sections of the text. The identified themes and codes were then described by using words and turning them into categories, looking for ways to reduce the total list of categories by grouping themes related to each other. The final decision was taken and themes, subthemes or categories were arranged alphabetically. The data were categorised according to the same themes and subthemes for interim analysis. Any irrelevant data were excluded and existing data were recorded. Consensus was reached with the co-coder via a virtual meeting to ensure that the results were a true reflection of the interviews.

### Measures of trustworthiness

*Credibility* was ensured through interviews lasting between 30 and 40 min of participants’ engagement. A voice recorder was used during the interview process to capture the data. *Transferability* was achieved by conducting interviews until data saturation was reached to ensure that no new information came forth. A full and thick description of research methods was provided. *Dependability* was achieved using a co-coder for data analysis to compare and reach consensus with data analysed by the researcher. *Confirmability* was achieved by using an independent co-coder during data analysis.

### Ethical considerations

Ethical approval to conduct the study was obtained from the University Ethics Committee (clearance number: NWU-00307-20-A1). Then, approval was obtained from other relevant authorities such as the DBE and all four school principals and school governing bodies. Participants were treated fairly and were informed that they could stop the interview at any time without any penalty if they wished so. All participants participated voluntarily and were not coerced. WhatsApp video calls were carried out in a private room, assuring confidentiality and anonymity.

## Results

### The participants

Participants’ demographic profiles were as follows: there were seven women (54%) and six men (46%). The age group of the study participants ranged from 31 to 35 years (46%), 50 to 55 years (23%), 40 to 45 years (23%) and 25 to 30 years (8%). The years of teaching experience ranged from 5 to 10 years (54%), 15 to 20 years (23%) and 25 to 30 years (23%). Lastly, the school representation was as follows: School A (23%), school B (31%), school C (23%) and school D (23%).

### Themes and sub-themes identified

The study yielded five themes and 17 sub-themes (see Table 1).

**TABLE 1:** Perceptions of the school management teams in managing pregnant learners in urban secondary schools.

Themes		Sub-themes
1.	Role of SMTs in managing pregnant learners at school	1.1. Identification of pregnant learners 1.2. Support 1.3. Monitoring of pregnancy
2.	Management of unplanned deliveries at school	2.1. Lack of delivery skills 2.2. Parental involvement 2.3. Policy issues
3.	Personal thoughts on managing pregnant learners	3.1. Negative feelings 3.2. Anxiety 3.3. Negative effects of learner pregnancy on educational progress
4.	Skills training requirements for educators to manage pregnant learners	4.1. Basic first-aid training 4.2. Counselling skills
5.	Coping mechanisms for SMTs	5.1. Presence of nurses at school 5.2. Active parental involvement 5.3. Reliable emergency services 5.4. Resources and infrastructure 5.5. Visible campaigns 5.6. Youth unfriendly health services

SMTs, school management teams.

#### Theme 1: Role of school management teams in managing pregnant learners at school

Managing pregnant learners at school is a task that forms part of the SMT roles. Three sub-themes emerged from this theme: identification of pregnant learners, support and monitoring of pregnancy.

**Sub-theme 1.1: Identification of pregnant learners:** Participants described the role of identifying pregnant learners as a primary responsibility of the SMT’s in the management of pregnant learners. Some participants mentioned that pregnant learners rarely report pregnancies on their own and they often conceal pregnancy by wearing baggy uniform until they discover them, which is usually when they are far along in pregnancy:

‘As SMTs, part of our duty is to identify the pregnant learners because most of them do not report the pregnancy until we identify them.’ (Participant 1, female, 50–55 years)

‘Our role is to identify pregnant learners, so that they can complete the necessary documents.’ (Participant 5, female, 31–35 years)

**Sub-theme 1.2: Support:** According to the MPMLP policy the SMTs must ensure that pregnant learners are not subjected to any form of discrimination, hate speech and harassment at school. Furthermore, they must communicate with the Department of Social Development (DoSD) about pregnant learners where applicable and facilitate in registering these learners for child support grants. Participants mentioned that they offer support through counselling to assess learner’s needs because most of the pregnant learners are from poor socio-economic backgrounds. Poverty is viewed as both a contributor and a consequence of early pregnancy (Cook 2021:27). In addition to support, the SMTs must ensure that the pregnant learner was not subjected to coerced sex. Chirinda et al. (2012) observed that gender imbalances restrict young girls’ sexual choices and many pregnancies result from coercive sex where girls are powerless to bargain for condom use:

‘I talk to them to assess the level of support they might need. Many learners here are from poor backgrounds too.’ (Participant 7, male, 31–35 years)

‘I counsel them … these learners often get nasty comments from other educators and other learners … imagine a teacher saying, “mothers are not allowed to make noise in my class… it’s not right.”’ (Participant 6, female, 31–35 years)

‘Talking to pregnant learners is important … we assess whether the pregnancy is not a result of coerced sexual intercourse… it’s bad.’ (Participant 13, male, 40–45 years)

**Sub-theme 1.3: Monitoring of pregnancy:** The SMTs’ monitoring of pregnancy includes providing pregnancy statistics to the district every month as required by the MPMLP. The SMTs use the pregnant learner’s clinic card to check how far they are in their pregnancy so that at the relevant time, the pregnant learners must be accompanied by their parents or guardians to school for support and monitoring:

‘We ask the pregnant learners to bring their clinic cards to school; we make copies so that we can monitor them and also for emergencies.’ (Participant 8, female, 50–60 years)

‘I ask the pregnant learner to bring their clinic card so that we can update our pregnancy register.’ (Participant 9, female, 31–35 years)

‘We ask for their clinic card so that we can check how far along they are because when they are 7–9 months, they must bring their parent with them to school.’ (Participant 11, female, 50–55 years)

### Theme 2: Management of unplanned deliveries at school

Pregnancies that happen prior to the age of 20 years bear more health risks, such as pregnancy-induced hypertension, pre-term delivery because of physiological immaturity and placenta abruption, postpartum haemorrhage for the mother (Panday et al. 2009; Wall-Wieler, Roos & Nickel 2016; WHO 2020). Unplanned deliveries carry a higher risk of complications for the new-born baby such as respiratory distress and hypothermia among other complications (Grovink & Sandoy 2018:18). Under this theme, the following sub-themes emerged: lack of delivery skills, parental involvement and policy issues.

**Sub-theme 2.1: Lack of delivery skills:** Unplanned delivery is described as ‘a birth of a neonate that occurs out-of-healthcare facility, without a midwife and optimal healthcare conditions’ (Javaudin et al. 2019:2). Participants acknowledged a lack of delivery skills to deliver a baby as they are not trained health care professionals. Participants mentioned that they did not feel confident to carry out the process of delivering of a baby. The MPMLP does not stipulate the process the SMTs should follow when unplanned deliveries occur:

‘I was once faced with that situation. All the SMT members ran away, and I was left alone as a male to attend to this learner … I was very scared as I didn’t know what to do while waiting for the ambulance.’ (Participant 4, male, 45–50 years)

‘I am not a nurse. I wouldn’t know what to do, I don’t have the necessary skills required to deliver a baby.’ (Participant 9, female, 31–35 years)

One participant had a different view:

‘Female teachers attend to the pregnant learner because it would not be appropriate for me as a male to attend to that kind of a situation.’ (Participant 3, male, 25–30 years)

**Sub-theme 2.2: Parental involvement:** Parents and guardians are required by the MPMLP to take the lead in working with the schools to support and monitor their pregnant children. Participants explained that most parents and guardians are hesitant to be involved because they are embarrassed and this shifts the responsibility to them. Parents and guardians are obligated to accompany and monitor their pregnant children from start of school day to the end when the pregnant learner is 7–9 months along:

‘Most parents are hesitant to come and monitor their children. I think because they are embarrassed. Others don’t care and they know that policy doesn’t allow us to chase pregnant learners from attending school.’ (Participant 4, male, 45–50 years)

‘I once phoned a parent to come to school for an emergency…she only came 5 h later to school and her child was already at the clinic.’ (Participant 1, female, 50–55 years)

**Sub-theme 2.3: Policy issues:** Participants stated that policy constraints that prevent them from touching or transporting pregnant learners in their private cars to the nearest clinics or hospitals cause them difficulties in doing what they believe is best for the pregnant learner. However, participants reported going beyond what policy is required to help learners and said:

‘I take the risk and use parental instincts; the clinic is just 2 km away from the school. I have done this twice….’ (Participant 2, male, 45–50 years)

‘I transported a pregnant learner before; an ambulance was called in the morning…it did not arrive. I had to take that learner to the clinic after school.’ (Participant 1, female, 50–55 years)

#### Theme 3: Personal thoughts on managing pregnant learners

Three sub-themes emerged under this theme: negative feelings, anxiety and negative effects of learner pregnancy on educational progress.

**Sub-theme 3.1: Negative feelings;** Participants shared similar negative feelings towards the management of pregnant learners in school:

‘Personally … it’s unfair on us as educators. I am not a gynaecologist; my job is to be an educator…. managing pregnant learners is not part of our modules when we study education.’ (Participant 9, female, 31–35 years)

‘I believe that sex before marriage is a sin. So, I feel like the parents leave this big responsibility to us. They don’t engage with their children when it comes to sex education. Other parents are still telling their kids that kids come from an airplane. Just imagine huh!’ (Participant 13, male, 40–45 years)

‘I feel that this is an extra-burden to us educators, especially the SMTs, because we go beyond our scope of practice. We get little or no support from parents/guardians.’ (Participant 11, female, 50–55 years)

‘I once had to invigilate just one learner, a matriculant who was in labour ward in hospital, I had to sit there for 3 h for her to write the exam, imagine sitting on the hospital benches… this government is too lenient.’ (Participant 8, female, 50–60 years)

**Sub-theme 3.2: Anxiety:** Some participants mentioned that they constantly have anxiety resulting from past traumatic experiences of pregnant learners:

‘I am still traumatised by an incident we once had here at school, where a learner went to the girl’s bathroom and she died there. It was found that she had done an illegal abortion.’ (Participant 10, female, 31–35 years)

‘I was traumatised by the amount of blood I saw that day. As a result, I don’t eat liver anymore.’ (Participant 4, male, 45–50 years)

**Sub-theme 3.3: Negative effects of learner pregnancy on educational progress:** According to the SA, DBE (2007:6), parents must ensure that as far as it is possible their pregnant children receive assignments and class tasks throughout the length of absence from school and ensure that the assignments and tasks are brought back to school for assessment. The SMTs mentioned that despite having catch-up programmes for pregnant learners to ensure they do not miss academic learning while they are absent from school, most pregnant learners perform poorly. Even though pregnant learners are allowed to attend school during pregnancy, more often they detach, causing quality of their learning and educational experience to be poor (UNESCO 2017:12). Participants felt discouraged by the pregnant learners’ educational progress, and they viewed pregnancy as having a negative impact on education because of absenteeism, which causes them to fall behind with schoolwork and projects and in turn leads to dropping out of school. Participants added that other learners drop out of school because they come from child-headed homes, once they give birth they do not come back to school because they do not have child minders:

‘You see these pregnant learners are constantly absent from school because they are sick, they go for their clinic follow-ups, they give birth, after birth they must take the baby for immunisation, they must also sort out the birth certificates for the babies so that they can apply for the social grant.’ (Participant 10, female, 31–35 years)

‘Other learners drop out of school because there is no one to look after the baby at home… this is not good for our school academic performances.’ (Participant 2, male, 45–50 years)

‘Personally, I think it’s wrong for learners to fall pregnant while still at school. They are jeopardizing their future as most of these learners come from poor family backgrounds.’ (Participant 5, female, 31–35 years)

#### Theme 4: Skills training requirements for educators to manage pregnant learners

Under this theme, two sub-themes emerged, namely, basic first-aid training and counselling skills.

**Sub-theme 4.1: Basic first-aid training:** The study participants suggested that basic first-aid training may be required for SMTs and educators to handle pregnant learners and other situations at school:

‘I feel that as the government gave us this extra job, they should at least provide us with first-aid training, sometimes learners faint and we don’t know how to assist them.’ (Participant 12, male, 31–35 years)

‘I don’t need any kind of training when it comes to pregnant learners … if I wanted to be a nurse, I would have studied nursing you see… but my passion is education.’ (Participant 9, female, 31–35 years)

**Sub-theme 4.2: Counselling skills:** Some participants proposed counselling skills training to assist educators to handle pregnant learners at school:

‘I feel that all educators, not just the SMTs, must be provided with basic counselling skills, because some educators have prejudice when it comes to pregnant learners. They just pass the buck to us SMTs.’ (Participant 5, female, 31–35 years)

#### Theme 5: Coping mechanisms for school management teams

Six sub-themes emerged under this theme: presence of nurses at school; active parental involvement; reliable emergency services; resources and infrastructure; visible campaigns and youth friendly health services.

**Sub-theme 5.1: Presence of nurses at school:** Participants expressed the importance of the presence of nurses in the schools to assist with pregnant learners:

‘It would mean a great deal to us if we can have nurses allocated to our schools to assist us. They can be here on a weekly basis to cover all the schools around.’ (Participant 1, female, 50–55 years)

‘If the government can send nurses to school to attend to the pregnant learners, I would be relieved… like I would concentrate on my primary role of being an educator.’ (Participant 9, female, 31–35 years)

**Sub-theme 5.2: Active parental involvement:** Participants agreed that active parental support and involvement were important in managing pregnant learners:

‘Parents should be key role players. Girls and boys should be advised the same, we must stop the whole notion of saying when we start early to teach children about sex, we are encouraging them to have sex.’ (Participant 12, male, 31–35 years)

‘We need parents to be very active and supportive in this. Sex education must be started at home by the parents then we can be the secondary source.’ (Participant 6, female, 31–35 years)

**Sub-theme 5.3: Reliable emergency services:** Some participants felt that the ambulance services were not quick enough to respond to emergencies regarding pregnant learners:

‘Our ambulance services do not respond in time… we called an ambulance at 11:00; it only arrived at 17:00.’ (Participant 4, male, 45–50 years)

‘The ambulance services are failing us; the ambulance only arrived 3 h later, when the learner already delivered the baby.’ (Participant 8, female, 50–60 years)

**Sub-theme 5.4: Resources and infrastructure:** Participants felt that there are specific resources and infrastructure needed for pregnant learners, for example, tables, chairs and school uniforms to cater for their changed needs:

‘As the government allows the pregnant learners to attend full-time, they must provide proper study desks, because my classroom is a lab, imagine sitting the whole day on a bar stool being 8 months pregnant …and they also don’t fit on the normal desk.’ (Participant 1, female, 50–55 years)

‘Government must also think of other uniform for pregnant learners…the things we see here, learners squeezing themselves in school tunics.’ (Participant 11, female, 50–55 years)

**Sub-theme 5.5: Visible campaigns:** Participants mentioned that there is a need of visible campaigns that specifically focus on preventing teenage pregnancy for learners:

‘There is a lack of visible campaigns when it comes to the prevention of teenage pregnancy here in South Africa. Have you seen a billboard anywhere with a pregnant teenager except in clinics or have you ever seen a TV advert addressing teenage pregnancy? The campaigns must be like those of HIV/AIDS, they must be everywhere.’ (Participant 7, male, 31–35 years)

**Sub-theme 5.6: Youth unfriendly health services:** Participants mentioned that many learners are doubtful about using health centres because of the poor reputation and bad attitudes of health care workers who must provide family planning or care to pregnant learners:

‘Most learners are skeptical to use community health centres around them because of the attitude they get from healthcare providers. One learner told me that she went to the clinic and was turned back. She was told that she’s too young to be seeking family planning services, she must concentrate on school.’ (Participant 11, female, 50–55 years)

‘Healthcare service providers in our communities do not have a good reputation when it comes to public service, that’s why the teenagers’ resort to seeking “back street abortions.”’ (Participant 9, female, 31–35 years)

## Discussion

Teenage pregnancy is a global complex issue (Hadley 2020:388). Factors contributing to learner pregnancies are but not limited to poor socio-economic background, gender-based violence, peer pressure, curiosity resulting in unsafe sex, poor sex education (especially from parents), poor family planning and boredom. The reviewed literature confirms this (Chirinda et al. 2012; Khapagawani & Kalipeni 2017; Panday et al. 2009; South Africa, Department of Social Development 2014; UNESCO 2017). The SMTs are responsible for identifying pregnant learners at schools as these learners do not report and often conceal the pregnancies until they are identified. Late bookings to antenatal care have a negative impact, especially for preventing mother-to-child transmission because teenagers are more at risk of acquiring human immunodeficiency virus (HIV) and acquired immunodeficiency syndrome (AIDS) infection (United Nations International Children’s Emergency Fund 2018 [2021]).

The SMTs are of the opinion that the DBE should engage with them when amending or formulating new policies regarding the management of pregnant learners at school as the current MPMLP (SA, DBE 2007) only outlines that pregnant learners must be made aware that there are no health professionals on the school premises. However, it does not outline the procedure to be followed by the SMTs in the case of unplanned delivery happening at school. Furthermore, the SMTs are not allowed to transport pregnant learners in their private vehicles to the nearest clinics or hospital, instead they must wait for the Emergency Medical Rescue Services (EMRS). A study carried out by Senekal and Vincent-Lambert (2021) revealed that EMRS function under resource-constrained conditions in SA. This is evident by the delay in turn-around times when contacted for assistance by the SMTs. Training skills for counselling and basic first-aid training were highlighted by the SMTs. The DoH and the DBE should work together to provide basic counselling skills and first-aid training not only to the SMTs but also to all educators, as this could benefit not only pregnant learners but also all learners at large. The SMTs believe that having an allocated nurse in school on a weekly basis to offer services such as family planning, antenatal follow-ups and other services could be beneficial for learners and educators. Pregnant learners will not have to miss the whole day of schooling for antenatal appointments, and this will also allay their constant feelings of nervousness when they have pregnant learners in the class. This position is also supported by Goal 4 of the United Nations’ Sustainable Development Goals (SDGs), which is to ensure inclusive and equitable quality education and promote lifelong learning opportunities for all. This can also be carried out by ensuring that pregnant and parenting girls continue their education within a safe school environment that is gender equitable and free from stigma and discrimination as this will increase educational retention and attainment by 2030 (United Nations Development Programme 2015). Although learners are allowed to continue with their schooling during pregnancy, the SMTs believe that government should look at upgrading the infrastructure of schools to cater for their changed needs such as provision of study desks that can comfortably accomodate pregnant learners. Pregnant learners also find themselves restricted in their school uniforms as they progress in their pregnancy journey. A need for visible teenage pregnancy campaign was highlighted by the participants. The campaigns could be carried out by advertising teenage pregnancy on billboards, TV and radio advertising could also reach a bigger audience and launching campaigns in schools could promote youth-friendly services offered at PHC level. The recommendations of youth-friendly healthcare services by the participants are in line with what the World Health Organization (2011) also recommends, that is, ensuring privacy, offering convenient operational hours, respect for youth by healthcare providers, offering a range of contraceptives and reducing waiting times (United Nations Population Fund 2015).

## Conclusion

The results of this study confirm that teenage pregnancy is a multidimensional problem that requires a collaboration between DBE, DoH and DoSD to curb the rates of teenage pregnancy. The SMTs were able to describe how they perceived the management of pregnant learners, which was a challenge that required them to multitask between being educators offering normal lessons and catch-up lessons to pregnant learners who miss school; and being parents offering emotional support to those in need. In addition offering medical support for which they are not qualified. The SMTs added that despite having catch-up programmes for pregnant learners to ensure they do not miss academic learning while they are absent from school, most pregnant learners perform poorly, are demotivated and end up dropping out of school. Poor socio-economic backgrounds such as child-headed homes and parents’ unemployment were cited as contributing factors to school dropout rates, which also have a negative impact on the overall academic performances of schools. This study was conducted in one district of Gauteng province and was limited to SMTs’ perceptions; therefore, the results cannot be generalised to other secondary schools in the province.
